# Supplementary material for: Resolving the full spectrum of human genome variation using Linked-Reads
Source: Genome Res. 2019 Apr;29(4):635–45. doi: 10.1101/gr.234443.118 (PMC6442396; doi:10.1101/gr.234443.118)
Supplement: Supplemental Material [file supp_gr.234443.118_Supplemental_longranger-2.2.zip › longranger-2.2.tar.bz2!/lr2.2-public/lib/stan/cmdstan/stan/doc/api/html/agrad_2rev_2error__handling_2matrix_2check__pos__definite_8hpp.html]

Stan: src/stan/agrad/rev/error\_handling/matrix/check\_pos\_definite.hpp File Reference


|  |  |
| --- | --- |
|  | Stan  2.5.0  probability, sampling & optimization |


- Main Page
- Related Pages
- Namespaces
- Classes
- Files

- File List
- File Members

All Classes Namespaces Files Functions Variables Typedefs Enumerations Enumerator Friends Macros Pages

- src
- stan
- agrad
- rev
- error\_handling
- matrix

Namespaces |
Functions

check\_pos\_definite.hpp File Reference

`#include <stan/agrad/rev/numeric_limits.hpp>`  
`#include <stan/agrad/rev/var.hpp>`  
`#include <stan/math/error_handling/matrix/check_pos_definite.hpp>`  
`#include <stan/math/matrix/Eigen.hpp>`  
`#include <stan/math/matrix/meta/index_type.hpp>`

Go to the source code of this file.

|  |  |
| --- | --- |
| Namespaces | |
|  | stan |
|  | Probability, optimization and sampling library. |
|  | |
|  | stan::agrad |
|  | Function gradients via reverse-mode automatic differentiation. |
|  | |

|  |  |
| --- | --- |
| Functions | |
| template<typename T\_result , class Policy > | |
| bool | stan::agrad::check\_pos\_definite (const char \*function, const Eigen::Matrix< var, Eigen::Dynamic, Eigen::Dynamic > &y, const char \*name, T\_result \*result, const Policy &) |
|  | |


---

[ Stan Home Page ]

*© 2011–2014,
Stan Development Team.*
